# PGF_2α_-F-prostanoid receptor signalling via ADAMTS1 modulates epithelial cell invasion and endothelial cell function in endometrial cancer

**DOI:** 10.1186/1471-2407-10-488

**Published:** 2010-09-14

**Authors:** Margaret C Keightley, Kurt J Sales, Henry N Jabbour

**Affiliations:** 1 Medical Research Council Human Reproductive Sciences Unit, The Queen's Medical Research Institute, The University of Edinburgh, Edinburgh, EH16 4TJ, UK

## Abstract

**Background:**

An increase in cancer cell invasion and microvascular density is associated with a poorer prognosis for patients with endometrial cancer. In endometrial adenocarcinoma F-prostanoid (FP) receptor expression is elevated, along with its ligand prostaglandin (PG)F_2α_, where it regulates expression and secretion of a host of growth factors and chemokines involved in tumorigenesis. This study investigates the expression, regulation and role of a disintegrin and metalloproteinase with thrombospondin repeat 1 (ADAMTS1) in endometrial adenocarcinoma cells by PGF_2α _via the FP receptor.

**Methods:**

Human endometrium and adenocarcinoma tissues were obtained in accordance with Lothian Research Ethics Committee guidance with informed patient consent. Expression of ADAMTS1 mRNA and protein in tissues was determined by quantitative RT-PCR analysis and immunohistochemistry. Signal transduction pathways regulating ADAMTS1 expression in Ishikawa cells stably expressing the FP receptor to levels seen in endometrial cancer (FPS cells) were determined by quantitative RT-PCR analysis. In vitro invasion and proliferation assays were performed with FPS cells and human umbilical vein endothelial cells (HUVECs) using conditioned medium (CM) from PGF_2α_-treated FPS cells from which ADAMTS1 was immunoneutralised and/or recombinant ADAMTS1. The role of endothelial ADAMTS1 in endothelial cell proliferation was confirmed with RNA interference. The data in this study were analysed by T-test or ANOVA.

**Results:**

ADAMTS1 mRNA and protein expression is elevated in endometrial adenocarcinoma tissues compared with normal proliferative phase endometrium and is localised to the glandular and vascular cells. Using FPS cells, we show that PGF2α-FP signalling upregulates ADAMTS1 expression via a calmodulin-NFAT-dependent pathway and this promotes epithelial cell invasion through ECM and inhibits endothelial cell proliferation. Furthermore, we show that CM from FPS cells regulates endothelial cell ADAMTS1 expression in a rapid biphasic manner. Using RNA interference we show that endothelial cell ADAMTS1 also negatively regulates cellular proliferation.

**Conclusions:**

These data demonstrate elevated ADAMTS1 expression in endometrial adenocarcinoma. Furthermore we have highlighted a mechanism whereby FP receptor signalling regulates epithelial cell invasion and endothelial cell function via the PGF_2α_-FP receptor mediated induction of ADAMTS1.

## Background

Endometrial cancer is the most common gynaecological cancer among women in the Western world [1]. In 2009 the American Cancer Society estimated that there would be around 42,106 new cases of endometrial cancer resulting in approximately 7,780 deaths [1]. More than 80% of endometrial cancers are endometrial adenocarcinomas of the endometrioid type which originate from the endometrial lining [2]. Although the etiology of the disease is poorly understood, the occurrence of endometrial cancer has been linked to a variety of genetic and environmental influences, including age, obesity, diabetes and steroid hormones [2-4].

Our laboratory and others have demonstrated elevated expression of prostaglandin-endoperoxide synthase (PTGS, also called cyclooxygenase or COX)-2, biosynthesis of prostanoids [5,6], and elevated expression of prostanoid receptors [7], such as the F-prostanoid (FP or PTGFR) receptor in endometrial adenocarcinomas [7]. Moreover, we have shown that elevated PGF_2α_-FP receptor signalling in endometrial adenocarcinoma leads to upregulation of tumorigenic genes such as PTGS2 [8] and angiogenic genes such as FGF2 [9] and VEGF [10] which regulate vascular function in a paracrine manner [11]. FP receptor can also regulate the adhesiveness of endometrial adenocarcinoma cells to the extracellular matrix (ECM) via reorganisation of the actin cytoskeleton and activation of focal adhesion kinase [7,12]. These findings suggest that PGF_2α_-FP receptor signalling plays a multifactorial role in regulating endometrial adenocarcinoma by promoting an environment for angiogenesis and tissue remodelling to facilitate tumour growth. In addition to the regulation of cell architecture and adhesion to the ECM [12,13] the PTGS-PG axis has been shown to enhance the metastatic potential of tumour cells [14]. Indeed, we have shown that PGF_2α_, via the FP receptor, can enhance the motility of endometrial adenocarcinoma cells in vitro [12]. In endometrial cancer a more invasive phenotype and an increase in angiogenesis correlate with higher grade, poorly differentiated cancers [15]. Invasion is an essential cellular process facilitating tumour cell migration and metastasis. In breast and pancreatic cancer, the matrix metalloproteinase properties of a disintegrin and metalloprotease with a thrombospondin repeat (ADAMTS1), along with its anti-angiogenic role, have been shown to influence metastasis through the promotion of cellular migration and invasion [16,17]. ADAMTS1 was first identified as an inflammatory associated protein that anchored to the extracellular matrix via heparin dependent mechanisms [18,19]. ADAMTS1 expression is elevated in metastatic breast cancer [17] and pancreatic cancer, where its expression is associated with invasiveness and lymph node metastasis [16]. However, the expression and role of ADAMTS1 in endometrial adenocarcinoma has not been studied.

Here we investigated the expression and localisation of ADAMTS1 in endometrial adenocarcinoma and its regulation by PGF_2α _via the FP receptor. We found that ADAMTS1 expression was elevated in the glandular and vascular compartments in endometrial cancer compared with normal endometrium. Using in vitro model systems of Ishikawa endometrial epithelial cells stably expressing the FP receptor to levels seen in endometrial cancer (FPS cells) and human umbilical vein endothelial cells (HUVECs), we found that ADAMTS1 was regulated in epithelial cells via the PGF_2α_-FP receptor mediated activation of the calmodulin-NFAT pathway increasing epithelial cell invasion and negatively controlling endothelial cell proliferation.

## Methods

### Human Tissue

Endometrial cancer tissues and normal endometrial tissues were collected with ethical approval from Lothian Research Ethics Committee under ethics number LREC/1999/6/4 as detailed previously [20]. Written informed consent was obtained from all subjects prior to tissue collection. Endometrial cancer tissue was obtained from women undergoing surgery for removal of endometrial cancer and who had been pre-diagnosed on endometrial biopsy to have endometrial adenocarcinoma of the uterus of the endometrioid type. All patients were postmenopausal women with ages that ranged from 50-71 years of age and presented with complaint of postmenopausal bleeding. The median age of patients was 60.5 years. Cancer biopsies were assessed by a pathologist and assigned a grade, well differentiated (grade 1; n = 10), moderately differentiated (grade 2; n = 10) or poorly differentiated (grade 3; n = 10) as outlined in table 1. None of the carcinoma patients in this study were on hormone replacement therapy. Normal endometrium from the proliferative phase of the menstrual cycle (n = 10), was collected with an endometrial suction curette (Pipelle, Laboratoire CCD, France) from women undergoing surgery for gynecological procedures, including surgical sterilisation or abnormal uterine bleeding, and in whom histological examination of the endometrium was normal with no underlying endometrial pathology. The median age of these women was 30.5 years (range 21-39 yrs). Biopsies were dated according to stated last menstrual period (LMP) and confirmed by histological assessment [10] and hormone analysis as outlined in table 2. After collection, tissue was placed in RNAlater (Ambion) and stored at -70°C (for RNA extraction) or fixed in neutral buffered formalin and wax embedded (for immunohistochemical analysis).

**Table 1:** Tumour characteristics for well differentiated, moderately differentiated and poorly differentiated endometrial adenocarcinomas used in the study.

HISTOLOGY	FIGO STAGE	HISTOLOGY	FIGO STAGE
Well Differentiated	Ib	Poorly Differentiated	IIIa

Well Differentiated	Ib	Poorly Differentiated	Ia

Well Differentiated	Ia	Poorly Differentiated	IIIa

Well Differentiated	Ic	Poorly Differentiated	Ib

Well Differentiated	IIIa	Poorly Differentiated	Ib

Well Differentiated	Ib	Poorly Differentiated	IIIa

Well Differentiated	Ib	Poorly Differentiated	Ib

Well Differentiated	Ib	Poorly Differentiated	Ib

Well Differentiated	Ia	Poorly Differentiated	IIIa

Well Differentiated	Ia	Poorly Differentiated	IIb*

Mod Differentiated	Ib		

Mod Differentiated	Ic		

Mod Differentiated	IIb *		

Mod Differentiated	IIIa		

Mod Differentiated	Ic		

Mod Differentiated	Ic		

Mod Differentiated	Ic		

Mod Differentiated	Ia		

Mod Differentiated	IIb		

Mod Differentiated	Ib		

**Table 2:** Clinical parameters for normal endometrial samples from proliferative phase endometrium.

HISTOLOGY	PROGESTERONE nmol/L	ESTRADIOL pmol/L
Proliferative	1.78	400.00

Proliferative	4.80	204.00

Proliferative	3.12	731.00

Proliferative	2.32	1796.00

Proliferative	0.17	989.70

Proliferative	4.26	339.38

Proliferative	2.22	641.37

Proliferative	4.62	525.00

Proliferative	4.57	495.00

Proliferative	2.82	214.00


### Cell culture

Ishikawa cells stably expressing FP receptor (Ishikawa FPS cells) to levels observed in endometrial cancer were cultured in Dulbecco's Modified Eagle's Medium (DMEM, Invitrogen, Paisley, UK) with 10% foetal bovine serum (FBS) and 1% penicillin/streptomycin as described previously [10]. Human umbilical vein endothelial cells (HUVECs) (Lonza, Walkersville, USA) were cultured in Endothelial Basal Medium (EBM-2) with 2% FBS and growth supplements (VEGF, FGF, PGDF, IGF, EGF, ascorbic acid, heparin and gentamycin) subsequently referred to as Endothelial Growth Medium (EGM-2) (Lonza, Walkersville, USA). Concentrations of chemical inhibitors were determined by titration using the manufacturer's data sheet as a guide as described in our previous studies [10,12,20]. Cell viability in the presence of the chemical compounds used to inhibit specific signal transduction pathways was assessed using the CellTitre96AQueous One Solution™ (Promega, Southampton, UK).

### Conditioned medium

Conditioned medium (CM) was prepared as described previously [9]. Briefly, FPS cells were seeded at a density of 2 × 10^6 ^cells and allowed to adhere before serum-starvation for 24 hrs. Thereafter, cells were treated with 20mls of serum free DMEM containing 8.4 μM indomethacin in the presence of vehicle or 100nM PGF_2α _for 24 hrs to create vehicle conditioned medium (V CM) or PGF_2α _conditioned medium (P CM). Conditioned medium from three independent experiments was pooled, aliquoted and stored at -20 °C until required.

### ADAMTS1 immunoneutralisation

ADAMTS1 was immunoneutralised from PGF_2α _conditioned medium by overnight incubation on a rotar at 4°C, with 1 μg/ml ADAMTS1 antibody (ab28284, Abcam, Cambridge, UK) in accordance with our previous study [9]. Immunoglobulin (IgG) from the same species as the primary antibody was used as a control at the same concentration. Antibody concentration for immunoneutralisation was determined empirically by titration. The immune complex was removed by 4 hr incubation, on a rotar at 4°C, with 30 μl of a 50% protein G plus/protein A agarose mixture (Calbiochem, Nottingham, UK). Samples were centrifuged at 1500rpm for 5mins after which the immunoneutralised CM was aliquoted and stored at -20°C until use.

### Taqman quantitative RT-PCR

Taqman RT-PCR was performed as described previously using sequence specific primers and probes designed to span an intron [7,21,22]. RNA was extracted, reverse transcribed and RT-PCR performed using the ABI Prism 7900 as described previously [9,20]. Analysis of all samples was performed using the comparative CT method (ΔΔCT) and expressed relative to a positive RNA standard (cDNA obtained from pooled endometrial cDNA) included in all reactions. The expression of ADAMTS1 was normalized for RNA loading using ribosomal 18 S RNA as an internal standard in the same reaction. Where data are expressed as fold above control, the relative ΔΔCT value for the treatment group (or P CM/P CM and inhibitor) was divided by the ΔΔCT for the vehicle group (or V CM/V CM and inhibitor). Data are represented as mean ± SEM.

### Immunohistochemistry

ADAMTS1 protein expression was localized in endometrial adenocarcinoma tissues (n = 15) and proliferative phase endometrium (n = 5) by immunohistochemistry. Briefly, five-micron paraffin wax-embedded tissue sections were cut and mounted onto coated slides (TESPA, Sigma, Dorset, UK). Sections were dewaxed in xylene, rehydrated in graded ethanol and washed in water followed by TBS (50 mM Tris-HCl, 150 mM NaCl pH7.4) and blocked for endogenous endoperoxidase (1% H_2_O_2 _in methanol). Antigen retrieval was performed by pressure cooking for 2 minutes in 0.01 M sodium citrate pH6. Sections were blocked using 5% normal swine serum diluted in PBS with 5% BSA. Tissue sections were incubated with rabbit anti-human ADAMTS1 polyclonal antibody recognising the amino terminal end of ADAMTS1 (ab28284, Abcam) (1:100 dilution) overnight at 4°C. Control sections included the following: no primary antibody or rabbit IgG. After washing in TBS, sections were incubated with swine anti-rabbit biotinylated antibody (Dako), followed by streptavidin-horseradish peroxidase complex (Dako). Colour reaction was developed using 3'3 diaminobenzidine (Dako). Sections were counterstained in haematoxylin. Images were obtained on a Provis AX70 microscope (Olympus America Inc., NY, USA) using Canon EOS image capture software (Canon, Reigate, UK).

### Immunofluorescence

Dual immunofluorescence for ADAMTS1 and CD31 expression was performed as previously described [9]. Antigen retrieval was performed by pressure cooking for 2 minutes in 0.01 M sodium citrate pH6. Sections were blocked in 5% normal goat serum diluted in PBS with 5% BSA before incubation with ADAMTS1 antibody (1:600 dilution). Following overnight incubation at 4°C, sections were sequentially incubated with goat anti-rabbit biotinylated Fab (1:500 dilution) and then tyramide signal amplification kit (TSA Fluorescein System; 1:50 dilution; Perkin-Elmer). Sections were then microwaved in 0.01 M citrate buffer for 30 min and endogenous peroxidase blocked using hydrogen peroxide. Nonspecific binding was blocked with 5% normal goat serum. Thereafter sections were incubated with rabbit anti-human CD31 (1:1000 dilution, Sigma) at 4°C overnight. Sections were again incubated with goat anti-rabbit biotinylated Fab and tyramide signal amplification kit. Nuclei were counterstained using Dapi (1:1000 dilution, Molecular Probes). Sections were mounted in Permafluor (Immunotech-Coulter, Marseille, France) and visualised and photographed using a Carl Zeiss laser scanning microscope LSM510 (Jena, Germany).

### Invasion assay

Ishikawa FPS cell invasion was analysed using an 8 μm polycarbonate membrane Transwell insert (Corning, NY, USA). Membranes were coated with 20 μl of growth factor (GF)-reduced Matrigel (BD Biosciences, MA, USA) and incubated at 37°C for 30 min to allow thin layer gel formation. FPS cells (2 × 10^5 ^cells/well) were seeded on the membrane (upper chamber) in 500 μl serum free DMEM. In the lower chamber, 750 μl of V CM, P CM, P CM immunoneutralised with IgG or ADAMTS1, or recombinant ADAMTS1 (1nM or 10nM in serum free medium; R&D systems) were added. Serum free DMEM or complete DMEM were added to the lower chambers as controls. After 24 hrs incubation at 37°C in a 5% CO_2 _atmosphere, cell membranes were removed and cells were fixed for 30 min in 100% ice cold methanol. Non-migrated cells on the upper side of the membranes were removed with a cotton swab and membranes were stained with haematoxylin. Cells on the underside of the membranes were photographed (five fields per membrane at ×100 magnification) using an inverted microscope and camera (Axiovert 200, Carl Zeiss, Germany). Fold difference was determined by dividing the value obtained from P CM or IgG/ADAMTS1 treated cells by the value obtained from V CM or serum free treated cells. Data are represented as fold increase in invasion with V CM or serum free medium = 1 and are presented as mean ± SEM.

### Proliferation assay

HUVECs were seeded in 96-well plates at 3000 cells/well. Following attachment, cell medium was replaced with EBM1% for 3 hrs. Cells were then treated with V CM, P CM, P CM immunoneutralised with IgG or ADAMTS1 diluted 1:1 (v/v) with EBM1%. Treatments were replaced three times during the 96 hr incubation. Proliferation was determined using the CellTitre96AQueous One Solution™ (Promega) as per manufacturer's instructions. Fold difference was determined by dividing the absorbance obtained by P CM treated cells by the absorbance obtained by V CM treated cells. Data are represented as percentage increase in proliferation with V CM = 100% and are presented as mean ± SEM. Experiments performed in triplicate.

### ADAMTS1 siRNA transfection

ADAMTS1 siRNA was used to silence ADAMTS1 expression in HUVECs. ADAMTS1 Stealth siRNA duplexes consisting of three non-overlapping sequences which were commercially validated or control scrambled non-target siRNA was purchased from Invitrogen. Prior to the start of experiments the concentration of siRNA and transfection agent was optimised. Transfection efficiency for HUVECS was determined visually by transfection of cells with a green fluorescent protein-tagged expression vector to be approximately 40%. A scrambled non-target sequence of siRNA was used as a control. Silencing of ADAMTS1 expression was approximately 50% relative to scrambled control when using a pool of the three supplied stealth siRNA duplexes at equal ratio. HUVECs were seeded at 4 × 10^5^cells/25cm^2 ^flask. The next day, cells were transfected with 20nM control siRNA or ADAMTS1 siRNA using 5.7 μl siPORT Amine Transfection Agent (AppliedBiosystems). HUVECs were transfected with control siRNA and ADAMTS1 siRNA for 48hrs at 37°C in a 5% CO_2 _atmosphere after which cells were washed and treated for 12hrs with EGM media before performing proliferation assays.

### Statistical Analysis

The data in this study was analysed by T-test or ANOVA using Prism 4.0 (Graph Pad, San Diego, CA). A *P *value less than 0.05 was considered significant in all cases.

## Results

### ADAMTS1 expression is elevated in endometrial adenocarcinoma

**Figure 1:**
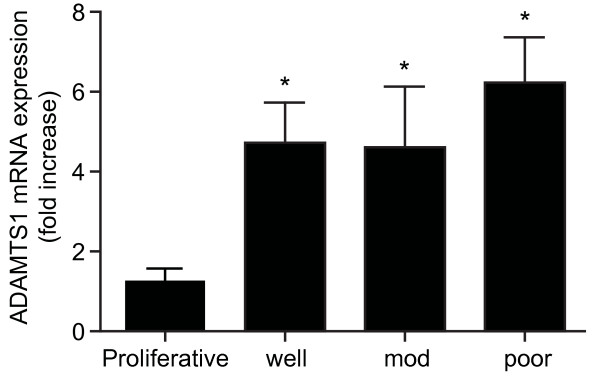
**ADAMTS1 mRNA expression in normal proliferative phase endometrium and endometrial adenocarcinoma**. Expression of ADAMTS1 mRNA in proliferative phase endometrium (n = 10) and well (n = 10), moderately (n = 10) and poorly differentiated (n = 10) endometrial adenocarcinoma as determined by quantitative RT-PCR analysis. * represents statistical significance compared to proliferative phase endometrium; P < 0.05. Data are represented as mean ± SEM.

We investigated the mRNA expression of ADAMTS1 in human endometrial adenocarcinoma and normal endometrium from the proliferative phase of the menstrual cycle by Taqman Quantitative RT-PCR analysis. We found that the expression of ADAMTS1 was elevated in all endometrial adenocarcinoma samples compared with proliferative phase endometrium (Figure 1; P < 0.05). There was no difference in the levels of ADAMTS1 expression irrespective of the grade or FIGO stage of endometrial adenocarcinoma, compared with proliferative phase endometrium.

### ADAMTS1 localisation in endometrial adenocarcinoma and normal endometrium

**Figure 2:**
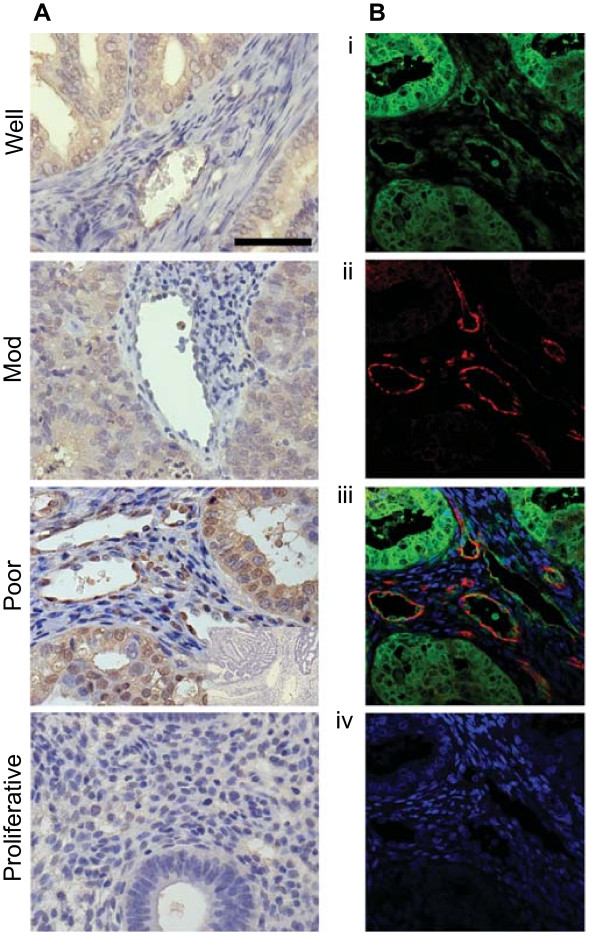
**Immunohistochemistry of ADAMTS1 in normal endometrium and endometrial adenocarcinoma**. **(A) **Localisation of the pattern of expression of ADAMTS1 in tissue samples of well, moderately and poorly differentiated endometrial adenocarcinoma and proliferative phase endometrium. Representative section of poorly differentiated endometrial adenocarcinoma incubated with IgG from the host species is shown as a negative control (inset). Black bar indicates 50 μm. **(B) **Dual immunofluorescence immunohistochemistry was used to co-localise (yellow channel; iii) the expression of ADAMTS1 (green channel; i) with the endothelial cell specific marker CD31 (red channel; ii) in a representative sample of poorly differentiated endometrial adenocarcinoma. Dapi was used as the nuclear counterstain (blue channel; iv).

Next we investigated the site of ADAMTS1 expression in well, moderately and poorly differentiated endometrial adenocarcinomas and proliferative phase endometrium. We observed strong immunoreactive staining in the glandular and vascular compartments of all well, moderately and poorly differentiated endometrial adenocarcinomas (Figure 2A; showing representative tissue sections). Under the same experimental conditions, minimal immunoreactivity was observed for ADAMTS1 in proliferative phase endometrium and no immunoreactivity was observed in control sections incubated with IgG from the host species (inset shown for poorly differentiated endometrial adenocarcinoma).

We confirmed the vascular localisation of ADAMTS1 in endometrial adenocarcinomas by dual immunofluorescence immunohistochemistry and confocal laser microscopy. ADAMTS1 expression (2Bi) co-localised (merged; 2Biii) with the endothelial cell marker CD31 in the blood vessels (Figure 2Bii**)**. Nuclear counterstain is shown in panel 2Biv. No immunoreactivity was observed in control tissue sections incubated with IgG from the host species (not shown).

### PGF_2α _-FP receptor signalling regulates ADAMTS1 expression

**Figure 3:**
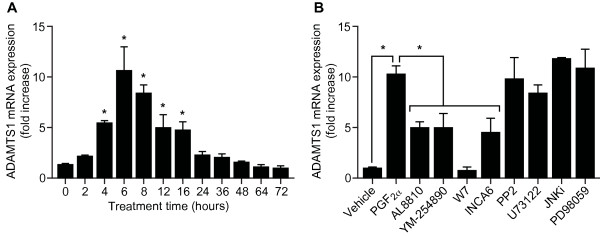
**ADAMTS1 mRNA expression in PGF_2α _treated FPS cells**. **(A) **FPS cells were treated with vehicle or 100nM PGF_2α _for 0, 2, 4, 6, 8, 12, 16, 24, 36, 48, 64 and 72 hrs and subjected to quantitative RT-PCR analysis for ADAMTS1. **(B) **FPS cells were treated for 8hrs with vehicle or 100nM PGF_2α _in the absence/presence of the FP receptor antagonist (AL8810), G_q/11 _inhibitor (YM-254890), calmodulin inhibitor (W7), NFAT inhibitor (INCA6), c-Src inhibitor (PP2), PLC inhibitor (U73122), JNK inhibitor (JNKi) or MEK inhibitor (PD98059) and mRNA expression of ADAMTS1 was determined by quantitative RT-PCR analysis. * represents statistical significance; P < 0.05. Data are represented as mean ± SEM from at least 3 independent experiments.

Since ADAMTS1 and FP receptor [7] are both expressed within the glandular and vascular compartments in endometrial adenocarcinoma, we investigated the potential regulation of ADAMTS1 in endometrial adenocarcinoma cells by PGF_2α _via the FP receptor using endometrial adenocarcinoma cells stably expressing the FP receptor to the levels observed in endometrial cancer (Ishikawa FPS cells; [10]). FPS cells were stimulated with vehicle or 100nM PGF_2α _for the times indicated in the figure legend. PGF_2α _stimulation resulted in a significant time-dependent increase in the expression of ADAMTS1 mRNA in FPS cells, which was maximal at 6-8hrs (Figure 3A, P < 0.05).

Consequently, the signalling pathway regulating the expression of ADAMTS1 was investigated using a panel of small molecule chemical inhibitors (Figure 3B). FPS cells were treated with vehicle or 100nM PGF_2α _in the absence or presence of the specific FP receptor antagonist AL8810 (50 μM), G_q/11 _inhibitor YM-254890 (1 μM), calmodulin inhibitor W7 (25 μM), NFAT inhibitor INCA6 (40 μM), c-Src inhibitor PP2 (10 μM), PLC inhibitor U73122 (10 μM), JNK-1 inhibitor JNKi (5 μM) or MEK inhibitor PD98059 (50 μM; Figure 3B). We found that ADAMTS1 mRNA expression was significantly elevated in response to PGF_2α _treatment after 8hrs of agonist stimulation. Co-incubation of FPS cells with PGF_2α _and AL8810, YM-254890, W7 and INCA6 significantly reduced the PGF_2α_-FP receptor mediated induction of ADAMTS1 (Figure 3B, P < 0.05). However, treatment of FPS cells with PGF_2α _and PP2, U73122, JNKi or PD98059 had no significant effect on ADAMTS1 mRNA expression in response to PGF_2α _treatment (Figure 3B). These data indicate that in FPS cells, the upregulation of ADAMTS1 involves PGF_2α_-FP signalling to G_q/11_, calmodulin and NFAT.

### PGF_2α_-FP signalling regulates epithelial cell invasion via ADAMTS1

**Figure 4:**
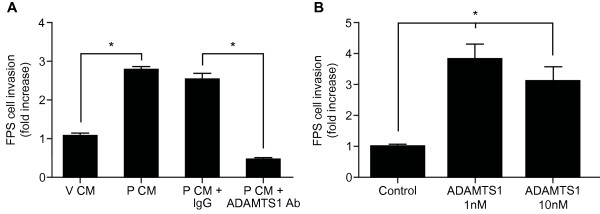
**The paracrine action of ADAMTS1 on FPS cell invasion**. **(A) **FPS cells were treated with V CM, P CM or P CM incubated with IgG or ADAMTS1 antibody for immunoneutralisation. Cell invasion through a monolayer of extracellular matrix was assessed after 24 hrs using a Boyden chamber assay. * represents statistical significance; P < 0.05. **(B) **FPS cells were treated with control (serum-free medium; SF) or SF medium with the addition of 1 or 10nM recombinant ADAMTS1 and cell invasion through a monolayer of extracellular matrix was assessed. * represents statistical significance of treated group relative to control; P < 0.05. Data are represented as mean ± SEM from at least 3 independent experiments.

ADAMTS1 has been shown to play a role in cancer cell metastasis [16,17]. We investigated whether PGF_2α _via the FP receptor could promote cell invasion of the ECM, a critical step in cancer cell metastases, via the induction of ADAMTS1. FPS cells were treated with vehicle (V) or 100nM PGF_2α _(P) for 24hrs to generate conditioned medium (CM). Using a modified Boyden chamber assay [23], we found that P CM significantly increased invasion of FPS cells through a layer of ECM compared to cells treated with control V CM (Figure 4A, P < 0.05). Furthermore, treatment of FPS cells with P CM in which ADAMTS1 had been immunoneutralised (P CM+ADAMTS1Ab), significantly inhibited FPS cell invasion compared with cells treated with P CM incubated with IgG (P CM+IgG) or P CM alone (Figure 4A, P < 0.05). We confirmed that ADAMTS1 enhanced FPS cell invasion using recombinant ADAMTS1 protein. We found that recombinant ADAMTS1 at both low (1nM) or high (10nM) doses significantly increased FPS cell invasion compared to control serum free medium (Figure 4B, P < 0.05). Furthermore there was no significant difference between ADAMTS1-induced FPS cells invasion at either concentration. This indicates that ADAMTS1 in the P CM, induced in response to PGF_2α_-FP receptor signalling, acts in a paracrine manner to promote FPS cell invasion through ECM.

### The paracrine action of ADAMTS1 in P CM-induced endothelial cell proliferation

**Figure 5:**
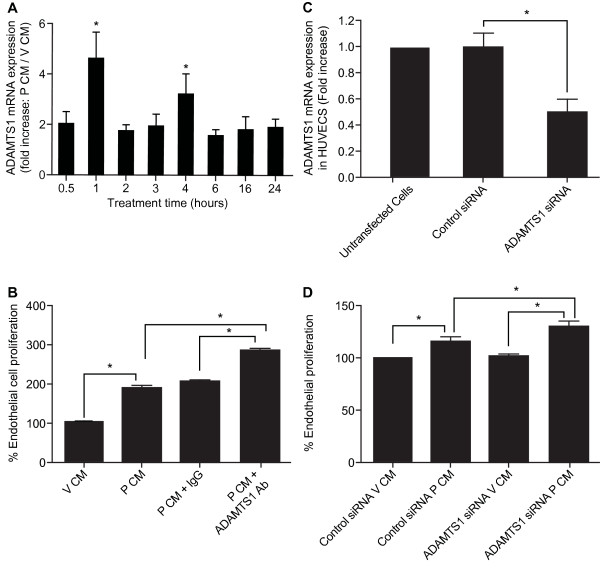
**ADAMTS1 regulates vascular function in vitro**. **(A) **HUVECs were treated with V CM or P CM for 0.5, 1, 2, 3, 4, 6, 16 and 24 hrs and ADAMTS1 mRNA expression was determined by quantitative RT-PCR analysis. **(B) **HUVECs were treated with V CM, P CM or P CM incubated with IgG or ADAMTS1 antibody for immunoneutralisation. Endothelial cell proliferation was assessed as described in the materials and methods. **(C) **HUVECs were transfected with control siRNA or ADAMTS1 siRNA for 48hrs or left untransfected for 48 hours. Thereafter ADAMTS1 mRNA expression was investigated by quantitative RT-PCR analysis. **(D) **HUVECs were transfected with control siRNA or ADAMTS1 siRNA for 48hrs. Subsequently, HUVECs were treated with V CM or P CM and proliferation assays were performed as described in the materials and methods. * represents statistical significance; P < 0.05. Data are represented as mean ± SEM from at least 3 independent experiments.

Since we found ADAMTS1 immunolocalised in the vasculature of endometrial adenocarcinoma (Figure 2B), we investigated the regulation of ADAMTS1 in endothelial cells in response to CM from FPS cells. We treated endothelial cells with V CM or P CM for the time indicated in the figure legend and investigated endothelial ADAMTS1 expression by quantitative RT-PCR analysis (Figure 5A). We found a dramatic elevation in endothelial ADAMTS1 mRNA expression at 1 and 4hrs of P CM treatment (Figure 5A; P < 0.05). We investigated the role of endothelial ADAMTS1 on endothelial cell proliferation as it has been described previously to be a potent anti-angiogenic factor [24]. We found that treatment of HUVECs with P CM significantly increased endothelial cell proliferation compared to V CM. Immunoneutralisation of ADAMTS1 from P CM (P CM+ADAMTS1Ab) further elevated endothelial cell proliferation compared with P CM alone or P CM incubated with IgG in place of neutralising antibody (P CM+IgG; Figure 5B, P < 0.05). Since ADAMTS1 is also expressed and produced in endothelial cells (Figure 2B) we investigated the role of endogenously produced endothelial ADAMTS1 on cellular proliferation using RNA interference. We used a cocktail of three commercially available validated siRNAs and found that we could suppress endogenous ADAMTS1 expression in HUVECs by approximately 50% when compared to a control non-target siRNA or untransfected cells (Figure 5C). Using this approach, we found that silencing of endothelial ADAMTS1 in HUVECs with ADAMTS1 siRNA prior to treatment with P CM also enhanced the proliferative effects compared with HUVECS transfected with control siRNA (Figure 5D, P < 0.05). These data suggest a dual mechanism for regulation of endothelial cell function by ADAMTS1 released from epithelial cells and endothelial cells.

## Discussion

Metastasis is one of the hallmarks of cancer, where neoplastic cells migrate away from the solid tumour, invade through ECM, and become dispersed around the body via the blood and lymphatics [25]. The process of metastasis is generally associated with poor prognosis and survival rates [26]. Although the mechanisms that regulate cancer metastasis are multiple, a link between the PTGS-prostaglandin pathway in breast and colon cancers has been established [14,27]. The specific molecular mechanisms and effector molecules which mediate metastasis, especially in the context of endometrial cancers are however poorly defined.

In this study, we investigated the expression, regulation and potential role of a disintegrin and metalloprotease with a thrombospondin repeat 1 (ADAMTS1) in endometrial adenocarcinomas. In breast and pancreatic cancer, ADAMTS1 has been shown to promote metastasis by enhancing cellular migration and invasion [16,17,28]. In the present study, we found that the expression of ADAMTS1 was upregulated coincident with the FP receptor [7] in well, moderately and poorly differentiated endometrial adenocarcinoma samples compared to normal endometrium from the proliferative phase of the menstrual cycle. Since the endometrium of post-menopausal women is no longer under normal hormonal control, is atrophic and often not attainable, we chose normal proliferative phase endometrium as our comparator. This is the phase of the menstrual cycle which exhibits rapid cellular proliferation, differentiation and tissue remodelling and is the phase of the menstrual cycle with the highest level of FP receptor expression [29].

We localised the site of expression of ADAMTS1 to the neoplastic epithelial and vascular cells of endometrial cancer tissues by immunohistochemistry and confocal laser microscopy. This pattern of expression in endometrial adenocarcinomas is similar to the expression profile for ADAMTS1 in secretory phase human endometrium as reported by Ng and colleagues, where expression is observed in the glandular epithelial and stromal cells [30]. In contrast to this latter study that reports readily detectable levels of ADAMTS1 throughout the menstrual cycle [30], in our study we found minimal immunoreactivity for ADAMTS1 in proliferative phase endometrium compared with the different grades of endometrial adenocarcinomas. Since the antibody concentration in our study was optimised for staining in the cancer tissues, we believe that the minimal immunostaining observed in the proliferative phase endometrium reflects the lower amount of ADAMTS1 protein in the normal endometrium compared with endometrial cancer and confirms our observations of differential mRNA expression in cancer and normal endometrial tissue presented in figure 1.

In order to investigate the regulation of ADAMTS1 in endometrial adenocarcinoma cells by the FP receptor and its potential role in endometrial cancer cell invasion, we used an endometrial cancer cell line stably expressing the FP receptor to the levels observed in endometrial adenocarcinoma (FPS cells) [10]. This in vitro approach has previously been used extensively to investigate PGF_2α_-FP receptor signaling [10,31] and robustly parallels the ex vivo effects of PGF_2α _on endometrial adenocarcinoma explants [9,10]. Using a panel of chemical inhibitors we found that ADAMTS1 expression was regulated by PGF_2α_-FP receptor signalling in FPS cells independently of the MAPK pathway via the Gq-mediated activation of calmodulin and NFAT.

When NFAT is dephosphorylated by effector signalling molecules, it translocates to the nucleus where it regulates target gene transcription [32]. NFAT is known to complex with other transcription factors, including activator protein 1 (AP1) within the transcriptome where it can regulate the expression of factors involved in cancer cell invasion [27,33].

In the present study we used a dual approach of treating FPS cells with FPS cell conditioned medium from which ADAMTS1 was immunoneutralised or treatment with recombinant ADAMTS1 protein. We have shown with both treatments that invasion of FPS cells through the ECM is mediated by ADAMTS1. These findings are similar to recent reports by Hatipoglu and colleagues and Krampert and colleagues, that demonstrate a role for ADAMTS1 in regulating cell movement [34,35]. Interestingly these latter studies report differential effects of ADAMTS1 depending on concentration and oxygen levels. For example, high concentrations of ADAMTS1 inhibits fibroblast migration by binding to and inactivating fibroblast growth factor- 2 under normoxic conditions and inhibits endothelial cell migration under hypoxic conditions [34,35]. However, in our study we found similar effects for ADAMTS1 in promoting FPS cell migration through a thin layer of ECM at both low (1nM) and high (10nM) concentrations under serum-free normoxic conditions. Although our study has not addressed the molecular mechanisms whereby ADAMTS1 regulates FPS cell invasion, several substrates have now been identified for ADAMTS1, including proteoglycans and aggrecan [36,37]. ADAMTS1 has been shown to cleave extracellular matrix proteins, such as syndecan 4 and semaphorin 3C, and to utilise metalloproteinase-dependent mechanisms to influence cell adhesion and migration [38-40]. Furthermore, upregulation of ADAMTS1 by ETS transcription factor gene (ERG) has been shown to contribute to an invasive phenotype in prostate cancer [41]. It is thus likely that ADAMTS1-mediated endometrial cell invasion is regulated via similar mechanisms following its release from epithelial cells in response to PGF_2α_-FP receptor signalling to NFAT.

In addition to regulating cellular invasion and metastasis, ADAMTS1 is also a potent anti-angiogenic factor [24]. Tumour angiogenesis is tightly regulated by a balance between pro-angiogenic and anti-angiogenic factors [42]. In our previous study we highlighted a role for the pro-angiogenic fibroblast growth factor-2, secreted from endometrial adenocarcinoma cells, in regulating endothelial network formation and proliferation [11]. Anti-angiogenic factors such as thrombospondin and endostatin have been shown to counteract the effects of pro-angiogenic factors to counterbalance endothelial cell proliferation in vitro and angiogenesis in vivo [43-47]. In accordance with this, we found that immunoneutralisation of ADAMTS1 from conditioned medium from PGF_2α_-treated FPS cells enhanced endothelial cell proliferation compared with conditioned medium alone, indicating that ADAMTS1 is an inhibitor of endothelial cell proliferation. Similar anti-angiogenic effects for ADAMTS1 have been reported in other systems. For example, ADAMTS1 expression in bovine aortic endothelial cells has been shown to inhibit endothelial cell proliferation and angiogenesis in vivo [24,48].

Furthermore, we found that endothelial cell expression of ADAMTS1 was also rapidly induced by conditioned medium from PGF_2α_-treated FPS cells in a biphasic manner, which was reciprocal to the expression pattern of the pro-angiogenic fibroblast growth factor 2 reported in our previous study [11]. This rapid time frame of induction of ADAMTS1 in endothelial cells, within 1 hour, is similar to recent reports for induction of this protein by hypoxia, indicating that it is likely to be an early response gene induced to tightly regulate endothelial cell proliferation [34]. Using RNA interference, we have shown that silencing ADAMTS1 expression in endothelial cells also enhanced endothelial cell proliferation. These data indicate a dual mechanism for the regulation of endothelial cell function by ADAMTS1 released from neoplastic epithelial cells and endothelial cells.

## Conclusion

**Figure 6:**
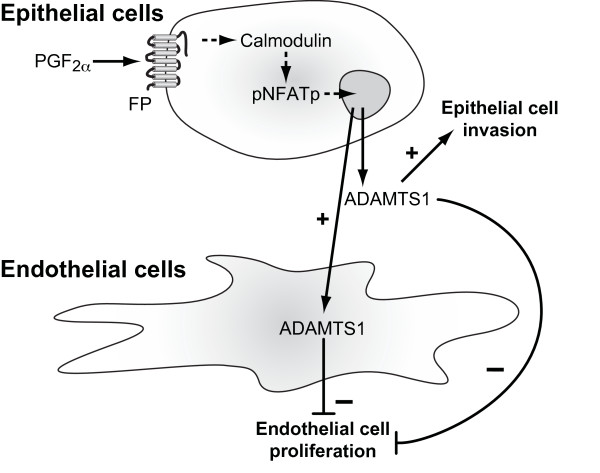
**Schematic diagram representing the role of ADAMTS1 in epithelial cell invasion and endothelial cell proliferation**. ADAMTS1 expression is elevated in the epithelial cells of endometrial adenocarcinoma by PGF_2α_-FP receptor signalling to the calmodulin-NFAT pathway. In turn ADAMTS1 acts in an autocrine/paracrine manner on tumour epithelial cells to regulate epithelial cell invasion through ECM. Moreover, ADAMTS1 secreted from epithelial cells acts in a paracrine manner on endothelial cells to inhibit cellular proliferation. In addition factors present in the conditioned medium from PGF_2α_- treated epithelial cells upregulates endothelial ADAMTS1 which in turn can act in an autocrine/paracrine manner to inhibit endothelial cell proliferation.

As summarised in Figure 6, this study presents novel data demonstrating that PGF_2α_-FP receptor signalling in endometrial adenocarcinoma cells upregulates ADAMTS1 expression via a G_q_-calmodulin-NFAT-dependent pathway. In turn ADAMTS1 acts in an autocrine/paracrine manner on tumour epithelial cells to regulate epithelial cell invasion through ECM. Moreover, it shows that ADAMTS1 acts in a paracrine manner on endothelial cells to inhibit cellular proliferation. In addition factors present in the conditioned medium from PGF_2α_- treated epithelial cells upregulate endothelial ADAMTS1 which in turn can act in an autocrine/paracrine manner to inhibit endothelial cell proliferation. Taken together our data highlight a mechanism whereby ADAMTS1, induced by PGF_2α_-FP signalling, regulates tumour cell invasion and endothelial cell proliferation in endometrial adenocarcinoma.

## Competing interests

MCK, KJS and HNJ would like to declare that there are no competing interests, financial or otherwise associated with this study.

## Authors' contributions

MCK carried out the experiments, participated in the design, statistical analysis, drafting and writing of the manuscript. KJS conceived the study, and participated in its design and coordination and helped to draft the manuscript. HNJ participated in the design and helped to draft the manuscript. All authors read and approved the final manuscript.

## Pre-publication history

The pre-publication history for this paper can be accessed here:

http://www.biomedcentral.com/1471-2407/10/488/prepub
